# Management of Congenital Midline Nasofrontal Masses: Case Report and Review of Literature

**DOI:** 10.1155/2015/159647

**Published:** 2015-03-25

**Authors:** A. C. Volck, G. A. Suárez, A. J. Tasman

**Affiliations:** Department of Otolaryngology, Head and Neck Surgery, Cantonal Hospital St. Gallen, Rorschacher Strasse 95, 9007 St. Gallen, Switzerland

## Abstract

Epidermoid cysts, dermoids, gliomas, and meningo-/encephaloceles are the most important differential diagnoses in congenital nasofrontal masses. Since they arise from an abnormal fusion during fetal development, intracranial extension of the lesion has to be ruled out radiologically before therapy. Dermoids are the most common entity. We report about a congenital epidermoid cyst of the glabella and nasion that had been growing over the last two years before presentation in a 24-year-old patient. We discuss radiological imaging and the different surgical approaches described in literature.

## 1. Introduction

Congenital midline masses of the face are uncommon, occurring in one out of 20.000–40.000 live births [1–3]. A male predominance has been reported [4]. The masses appear in the midline in the regions of embryonic fusion [5, 6]. These regions include the fonticulus frontalis, the prenasal space, and the foramen caecum. The fonticulus nasofrontalis is the space between the frontal and nasal bones. The prenasal space is between the nasal bones and the nasal capsule (the precursor of the septum and nasal cartilages). During normal fetal development these spaces are closed by fusion and ossification. The formation of dermoids, gliomas, and encephaloceles of the nose is considered to be the result of an abnormal development or incomplete fusion of the nasal processes. There are two theories for the pathogenesis of this entity. The so-called cranial theory suggests that, during the development of the frontal skull base, the dura mater retreating from the prenasal space adheres to the prenasal skin resulting in a sinus tract. Another theory, named superficial, suggests that abnormal congenital fusion at the nasal root with submucosal trapping of ectoderm between the two medial fusing nasal processes is responsible for the formation of a sinus or a cyst [7, 8]. Cysts containing skin adnexa are classified as dermoids. If an epithelium lined tract extends through the nasal septum to the foramen cecum, the developmental anomaly is classified as sinus or fistula [9].

## 2. Case Presentation

A 24-year-old patient presented to the Department of ENT, Head and Neck Surgery of the Cantonal Hospital St. Gallen, Switzerland, complaining of a nasofrontal mass existing since birth but slowly growing for the last 2 years. Other symptoms included a nonspecific frontal headache. There was no history of trauma, visual impairment, rhinorrhea, epistaxis, or hyposmia.

**Figure 1:**
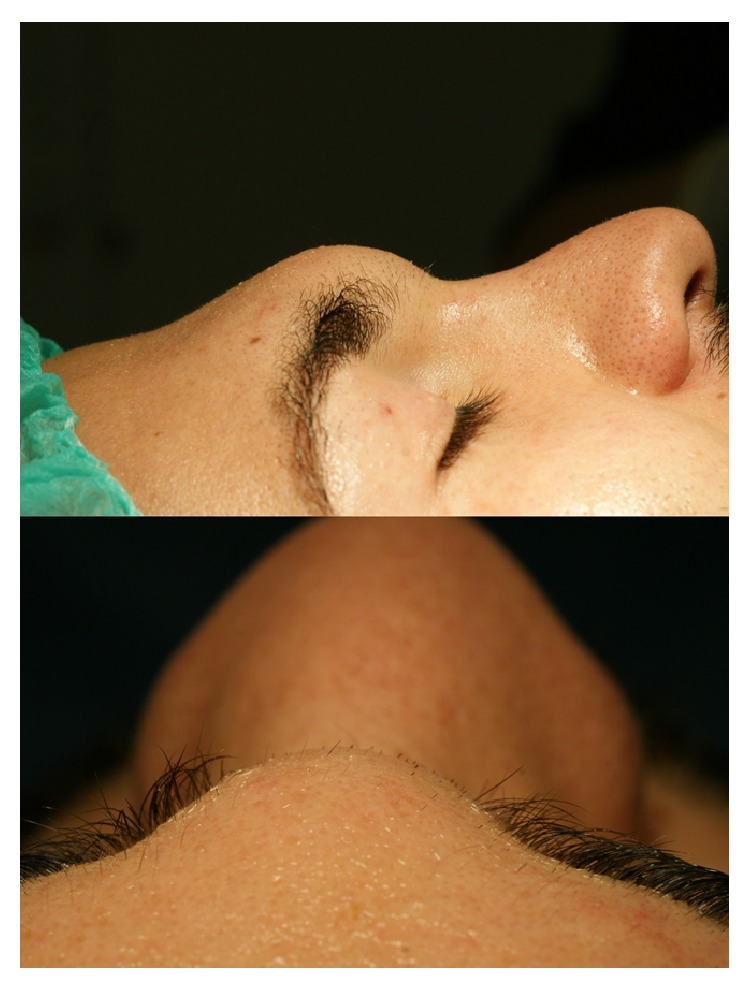
Preoperative pictures showing the mass at the glabellar area.

**Figure 2:**
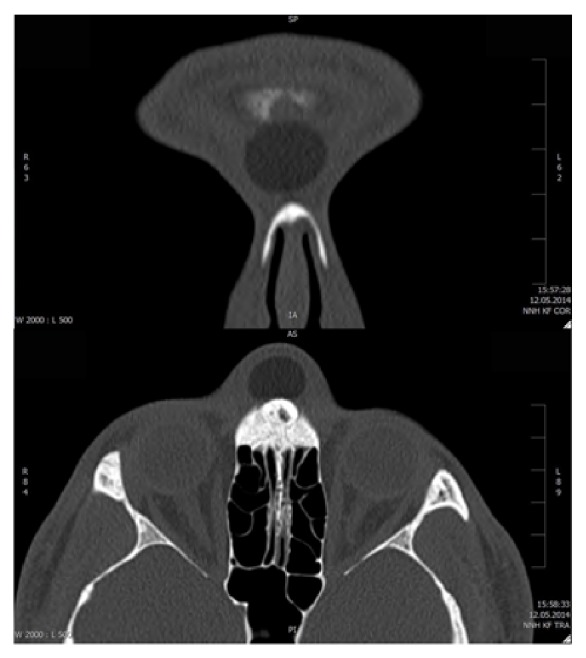
Coronal and axial CT images of the mass.

Clinical examination revealed a painless mass in glabellar area of approximately 2 cm in diameter (Figure 1). The mass was mobile over the nasal root and glabella without fixation of the overlying skin which appeared normal upon inspection. Nasal endoscopy revealed a normal endonasal anatomy. A CT scan showed a 2 × 1.3 × 2.2 cm mass located in the glabellar area. The mass appeared to be pushing in and thinning out the anterior wall of the frontal sinus. The posterior wall of the frontal sinus and the outflow tract appeared normal (Figure 2).

**Figure 3:**
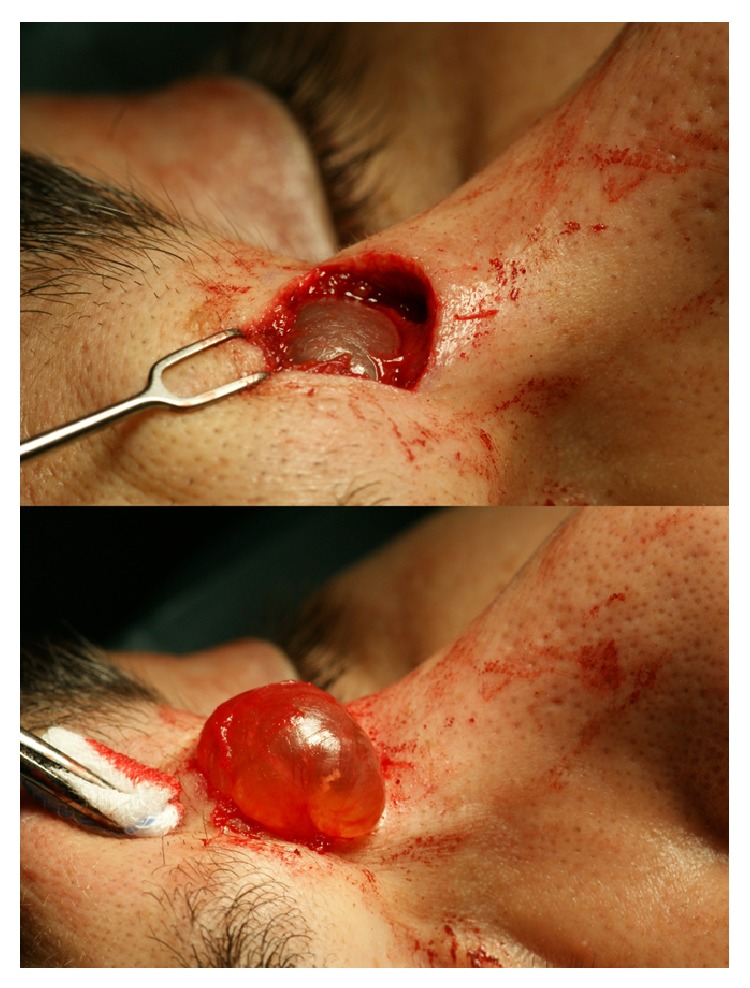
Intraoperative view showing the exposed cyst before removal.

Surgical excision was performed under local anesthesia through an incision along the relaxed skin tension lines (RSTL) in the glabella; blunt dissection revealed a cystic mass that did not infiltrate surrounding tissues (Figure 3).

Histological examination showed a cystic mass with an epithelial lining and macrophages consistent with a nasofrontal epidermoid cyst.

## 3. Discussion

### 3.1. Clinical Presentation

The congenital manifestation of the mass and growth during an infection is indicative of a congenital nasofrontal cyst or dermoid. Among the cystic lesions, dermoids are more prevalent than epidermoid cysts in children (58.9% versus 13.3%) [10]. An autosomal dominant inheritance has been reported in some families [11]. However, our patient reported not knowing the occurrence of similar masses in his family. Simultaneous congenital abnormalities such as cleft deformities, hydrocephalus, or aural atresia have been reported up to 41% of the cases. These findings double the risk of an intracranial extension when present [12]. When the mass originates from the neuroectoderm, encephaloceles, meningoceles, or meningoencephaloceles develop.

Nasofrontal dermoid cysts typically present as a mass with a small pore along the dorsal surface of the nose [13]. This is not the case in epidermoid cysts. Masses are often noticeable at birth gaining size over time with recurrent infections. Until the mass started growing visibly in our patient, it caused no discomfort. Medical consultation is typically sought for cosmetic concerns, recurrent infections, or pain [8]. Recurrent meningitis, not present in our patient, is rarely seen and should always raise suspicion of an intracranial sinus tract.

### 3.2. Differential Diagnoses

True dermoids and dermoid sinus or fistula, encephaloceles, meningoceles, and gliomas are the most important differential diagnoses of epidermoid cysts since they also present as a midline mass in the newborn [14]. Other congenital masses in this region include hemangiomas and teratomas. Patient's history regarding the onset and pattern of growth helps distinguishing acquired masses such as mucoceles and malignant tumours such as sarcoma.

### 3.3. Diagnosis

Radiological imaging often entails a CT scan as the primary investigation although ultrasound has reportedly been used [12]. Axial, sagittal, and coronal views are helpful to determine accurately the size and extent of the lesion as well as the integrity of adjacent bony structures. MRI scan is recommended to rule out intracranial extension or sinus tracts which can be found in 20–31% of the cases especially if the CT scan shows an enlarged Foramen caecum or a bifid Crista galli [12, 15, 16]. One study recommended MRI as sole examination, considering it to be the most cost effective and accurate evaluation [15]. Another advantage of the MRI over a CT scan is the avoidance of radiation since the majority of patients are children or young adolescents. In contrast to hemangiomas, dermoids are avascular and are not enhanced when applying contrast [17].

In the patient presented here, a CT scan was available and sonography was indicative of a cystic lesion obviating the need for an additional MRI scan.

When a dermoid, glioma, or encephalocele is suspected a biopsy may only be considered after an intracranial extension has been ruled out because of the risk of causing meningitis or CSF leak. An open biopsy of dermoid cysts is not recommended because of the increased risk of recurrence. Fine needle aspiration cytology is advocated by some for ruling out malignancy before any therapy is conducted, but the need for such investigation is controversial [4].

### 3.4. Therapy

Treatment of choice is the complete surgical excision preserving the cyst wall. Recurrence rates ranging from 50 to 100% have been described when the cyst wall had been damaged during surgery [15]. Dermoid cysts with or without intracranial extension have a risk of local recurrent infections and a high risk of CNS infection, in cases with an intracranial extension. Clinical observation seems to be a viable option after malignancy and intracranial involvement has been ruled out in patients without cosmetic impairment. In the case of a noticeable cosmetic deformity without malignancy or intracranial extension, the choice between surgical treatment and clinical observation should be discussed and decided together with the patient.

#### 3.4.1. Surgical Approaches

Several surgical techniques have been described in the literature. Choosing the approach will mainly depend on the location, the size of the lesion, and the surgeon's preference and experience. The patient's cosmetic concerns must be taken into account when discussing treatment options. The external transfacial approach by means of a vertical or horizontal incision or a Lynch incision is a straightforward approach offering a good exposure and facilitating complete removal of the lesion.

Transnasal approaches may offer superior cosmetic results but carry the risk of insufficient access to masses located cranially along the nasal bones or within the glabella. The transnasal approaches may be easier to perform in patients where skin laxity is favourable, such as the elder patient. However, it has been described in young patients as well [18]. Other external approaches such as the medial brow incision or a lateral rhinotomy provide good exposure at the cost of a potentially unfavourable scar.

Endoscopic techniques such as the endoscopic forehead approach allow good exposure of cranially located cysts while placing the incisions well camouflaged in the scalp. Drawbacks of this technique include wide undermining of the forehead and the risk of injury to neurovascular structures [19].

When an intracranial extension is present, a combined approach with a craniotomy may be required. Recently, less invasive techniques such as the transorbital neuroendoscopic approach (TONES) have been described with the orbital cavity serving as a corridor to the anterior skull base [20, 21].

**Figure 4:**
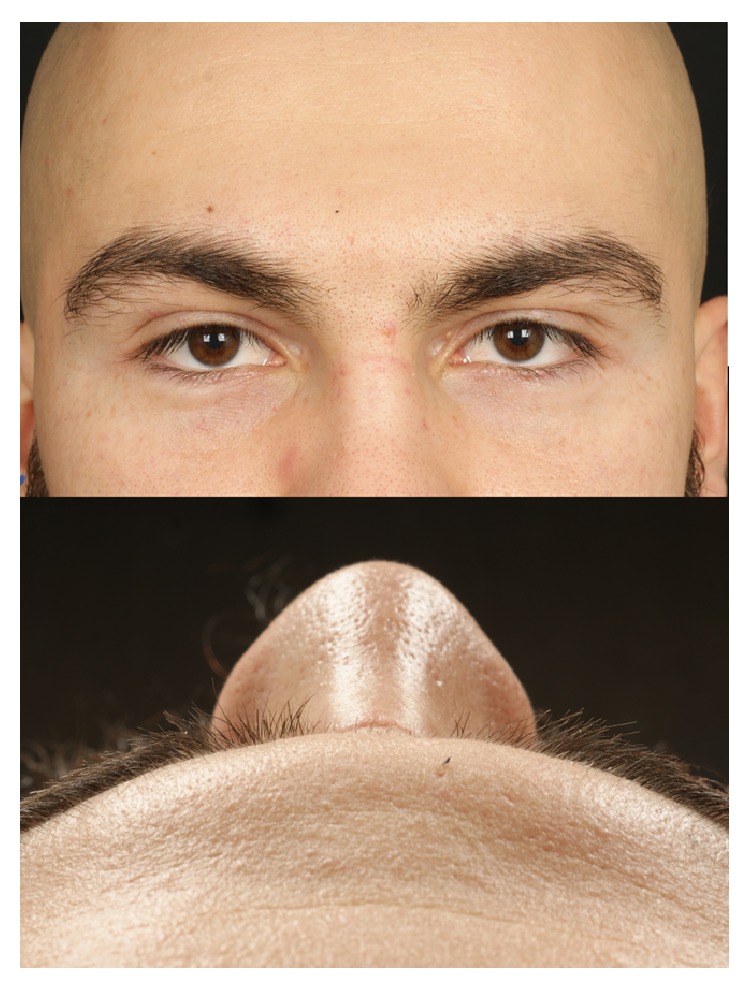
Six months after operation.

The primary concern in the case presented here was a complete surgical excision given the high recurrence rates reported when the cyst capsule is disrupted [15]. In spite of the cosmetic advantages of the endoscopic forehead or rhinoplasty approaches [4, 8, 18], such procedures usually entail risks of general anaesthesia and higher costs and may be more time consuming. Nonetheless, it may be preferable to some patients when given the option. Using local anaesthesia and basic soft tissue techniques we were able to perform complete surgical excision. With the skin incision placed within the RSTL and the use of everting suture technique the cosmetic result (Figure 4) was considered acceptable by the patient [22].

## 4. Conclusion

In conclusion epidermoid cysts or dermoids are rare but important differential diagnoses in a patient with a congenital nasofrontal mass. For radiological imaging a MRI scan is recommended in children or if the CT scan raises suspicion of an intracranial extension. Most authors would recommend both for assessing intracranial extension and planning the surgical approach. Surgery is the treatment of choice although not always necessary, especially if there is no history of infections, suspected malignancy, or evidence of intracranial extent. Recent articles advocate endoscopic approaches for superior cosmetic outcome. However, they do not appear to have gained wide popularity. We demonstrate that the external approach resulted in minimal scarring while offering superior exposure and a safe removal of the mass.

## References

[1] Hughes G. B., Sharpino G., Hunt W., Tucker H. M. (1980). Management of the congenital midline nasal mass: a review. *Head & Neck Surgery*.

[2] Paller A. S., Pensler J. M., Tomita T. (1991). Nasal midline masses in infants and children. *Laryngoscope*.

[3] Hsieh Y.-Y., Hsueh S., Hsueh C. (2003). Pathological analysis of congenital cervical cysts in children: 20 Years of experience at Chang Gung Memorial Hospital. *Chang Gung Medical Journal*.

[4] Dutta M., Saha J., Biswas G., Chattopadhyay S., Sen I., Sinha R. (2013). Epidermoid cysts in head and neck: our experiences, with review of literature. *Indian Journal of Otolaryngology and Head and Neck Surgery*.

[5] Dabholkar J. P., Patole A. D., Sheth A. S., Saaj R. (2003). Congenital cystic lesions in head and neck. *Indian Journal of Otolaryngology and Head & Neck Surgery*.

[6] Görür K., Talas D. Ü., Özcan C. (2005). An unusual presentation of neck dermoid cyst. *European Archives of Oto-Rhino-Laryngology*.

[7] Myer C. M., Cotton R. T. (1983). Nasal obstruction in the pediatric patient. *Pediatrics*.

[8] Holzmann D., Huisman T. A. G. M., Holzmann P., Stoeckli S. J. (2007). Surgical approaches for nasal dermal sinus cysts. *Rhinology*.

[9] Sessions R. B. (1982). Nasal dermal sinuses—new concepts and explanations. *Laryngoscope*.

[10] Armon N., Shamay S., Maly A., Margulis A. (2010). Occurrence and characteristics of head cysts in children. *Eplasty*.

[11] Bratton C., Suskind D. L., Thomas T., Kluka E. A. (2001). Autosomal dominant familial frontonasal dermoid cysts: a mother and her identical twin daughters. *International Journal of Pediatric Otorhinolaryngology*.

[12] Denoyelle F., Ducroz V., Roger G., Garabedian E. N. (1997). Nasal dermoid sinus cysts in children. *Laryngoscope*.

[13] MacGregor F. B., Geddes N. K. (1993). Nasal dermoids: the significance of a midline punctum. *Archives of Disease in Childhood*.

[14] Haafiz A. B., Sharma R., Faillace W. J. (1995). Congenital midline nasofrontal mass. Two case reports with a clinical review. *Clinical Pediatrics (Phila)*.

[15] Bloom D. C., Carvalho D. S., Dory C., Brewster D. F., Wickersham J. K., Kearns D. B. (2002). Imaging and surgical approach of nasal dermoids. *International Journal of Pediatric Otorhinolaryngology*.

[16] Pensler J. M., Bauer B. S., Naidich T. P. (1988). Craniofacial dermoids. *Plastic & Reconstructive Surgery*.

[17] Szeremeta W., Parikh T. D., Widelitz J. S. (2007). Congenital nasal malformations. *Otolaryngologic Clinics of North America*.

[18] Turner J. H., Tunkel D. E., Boahene D. K. (2010). Endoscopic-assisted, closed rhinoplasty approach for excision of nasoglabellar dermoid cysts. *Laryngoscope*.

[19] Sadick H., Huber M., Perkins S. W. (2014). Endoscopic forehead approach for minimally invasive benign tumor excisions. *JAMA Facial Plastic Surgery*.

[20] Moe K. S., Bergeron C. M., Ellenbogen R. G. (2010). Transorbital neuroendoscopic surgery. *Neurosurgery*.

[21] Ciporen J. N., Moe K. S., Ramanathan D. (2010). Multiportal endoscopic approaches to the central skull base: a cadaveric study. *World Neurosurgery*.

[22] Rahman M., Jefferson N., Stewart D. A., Oliver R., Walsh W. R., Gianoutsos M. P. (2010). The histology of facial aesthetic subunits: implications for common nasal reconstructive procedures. *Journal of Plastic, Reconstructive and Aesthetic Surgery*.

